# Mastectomy for management of breast cancer in Ibadan, Nigeria

**DOI:** 10.1186/1471-2482-13-59

**Published:** 2013-12-19

**Authors:** Temidayo O Ogundiran, Omobolaji O Ayandipo, Adeyinka F Ademola, Clement A Adebamowo

**Affiliations:** 1 Division of Oncology, Department of Surgery, College of Medicine, University of Ibadan and University College Hospital, Ibadan, Nigeria; 2 Division of Oncology, Department of Surgery, University College Hospital, PMB 5116 Ibadan, Nigeria; 3 Institute of Human Virology, Abuja, Nigeria; 4 Department of Epidemiology and Preventive Medicine, University of Maryland, Maryland, USA

**Keywords:** Breast cancer, Mastectomy, Nigeria

## Abstract

**Background:**

Modified radical mastectomy remains the standard therapeutic surgical operation for breast cancer in most parts of the world. This retrospective study reviews mastectomy for management of breast cancer in a surgical oncology division over a ten year period.

**Methods:**

We reviewed the case records of consecutive breast cancer patients who underwent mastectomy at the Surgical Oncology Division, University College Hospital (UCH) Ibadan between November 1999 and October 2009.

**Results:**

Of the 1226 newly diagnosed breast cancer patients over the study period, 431 (35.2%) patients underwent mastectomy making an average of 43 mastectomies per year. Most patients were young women, premenopausal, had invasive ductal carcinoma and underwent modified radical mastectomy as the definitive surgical treatment. Prior to mastectomy, locally advanced tumors were down staged in about half of the patients that received neo-adjuvant combination chemotherapy. Surgical complication rate was low. The most frequent operative complication was seroma collection in six percent of patients. The average hospital stay was ten days and most patients were followed up at the surgical outpatients department for about two years post-surgery.

**Conclusions:**

There was low rate of mastectomy in this cohort which could partly be attributable to late presentation of many patients with inoperable local or metastatic tumors necessitating only palliative or terminal care. Tumor down-staging with neo-adjuvant chemotherapy enhanced surgical loco-regional tumor control in some patients. The overall morbidity and the rates of postoperative events were minimal. Long-term post-operative out-patients follow-up was not achieved as many patients were lost to follow up after two years of mastectomy.

## Background

Surgery is the oldest treatment modality for cancer [1]. Its role has evolved over time to include prevention, diagnosis, treatment and rehabilitation. Total colectomy for familial adenomatous polyposis coli, prophylactic mastectomy and oophorectomy in people with familial risks for breast and ovarian cancers are all examples of cancer prevention surgery. Endoscopic and open surgical biopsies enhance tissue retrieval for the all-important pathological diagnosis and characterization of solid tumors. For many solid cancers, local and regional control is achieved by total surgical extirpation, or sometimes partial debulking, of the primary tumorr and the adjoining lymph nodes. Surgery is also increasingly employed in the management of solitary cancer metastases and in post treatment reconstruction and rehabilitation.

The role of surgery in the management of breast cancer consists in diagnosis, loco-regional tumor control in form of mastectomy and axillary dissection, and breast reconstruction. Vascular access for adjuvant chemotherapy and other parenteral infusions is often facilitated through outpatient surgical procedures. Furthermore, in metastatic breast cancer, surgical procedures are indicated for draining pleural effusions, stabilization bone and spine fractures and excising solitary deposits in the brain among others.

The surgical treatment of breast cancer has gone through phases. The early conservative excision gave way to Halsted’s radical mastectomy and this, with its many modifications, became the traditional surgical treatment for over a century [2]. In modern times, the use of conservative surgery as part of multidisciplinary management of breast cancer is increasing throughout the world. Complete surgical removal of the breast with its local lymphatic drainage remains the preferred surgical treatment in many parts of the world, especially in low income countries (LICs) where breast conserving surgery is infrequently practiced. This is because of the late stage of presentation of most patients, the large size of tumors at diagnosis, and the aggressive nature of the disease in a predominantly young population of women [3,4]. Moreover, breast conserving surgery in LICs is faced with many challenges including logistics limitations such as paucity of radiotherapy services and inefficient follow-up programmes.

There have been many publications on the surgical management of breast cancer from developed countries but few from developing countries [5-8]. In this paper we present findings from a review of breast cancer patients who had mastectomy in our division over a ten year period.

## Methods

We reviewed the case records of consecutive breast cancer patients who underwent mastectomy at the Surgical Oncology Division, University College Hospital (UCH) Ibadan between November 1999 and October 2009. A consultant surgeon supervised surgical residents and interns to extract data from wards admission registers, theater records and the division’s operation register and histology reports of mastectomy specimens. The information from those documents was used to retrieve the patients’ case files from the hospital records department. Individual patient’s consent was not required for this study due to its retrospective nature and being a review of routine clinical activities. However, permission was sought from the Chairman, Medical Advisory Committee (CMAC) of the hospital who doubles statutorily as the Vice Chairman of the University of Ibadan and University College Hospital Research Ethics Committee before accessing the patients’ information at the medical records department.

We recorded the patients’ demographics, breast cancer diagnosis, treatment modalities and outcomes of care. All the patients had pathological confirmation of breast cancer diagnosis by either needle or open surgical biopsy. Pathological diagnosis consisted of the histological type in all the patients and immunohistochemistry in some of them. The staging investigations recorded were mainly plain chest radiograph and abdomino-pelvic ultrasonography, radionuclide bone scan was added in the latter three years of the study. The information obtained from the patients’ case files were recorded in Microsoft excels spreadsheet. Descriptive methods were used to characterize the patients demography, clinical presentation, stage, treatments offered including types of mastectomy done and the outcomes of surgical treatment. Statistical analysis was done using SPSS version15 and the results are presented in charts and graphs.

## Results

**Figure 1:**
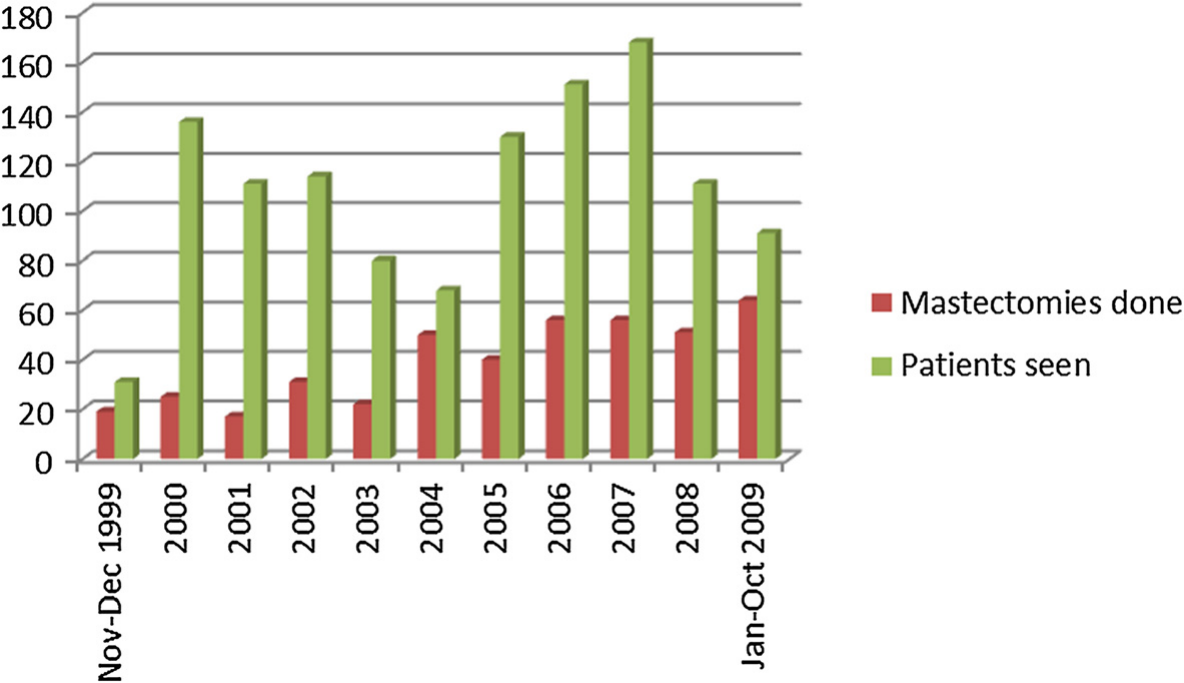
Numbers of breast cancer patients seen and mastectomies done per year in Surgical Oncology Division, UCH Ibadan in 1999–2009.

We retrieved the records of 1226 newly diagnosed breast cancer patients over the study period. Of these, 431 (35.2%) patients underwent mastectomy making an average of 43 mastectomies per year. Figure 1 shows the number of cases diagnosed and number that underwent mastectomy every year of the study. The case files of 354 patients constituting 82.1% of all mastectomies were available for review.

Table 1 shows the socio-demographic characteristics of the patients who had mastectomy. They were aged from 20 to 76 years with a mean (SD) of 47.4(11.3) years and a median of 43 years. As expected, the majority of patients (340, 96.0%), were females. Most were Yorubas (258, 73.0%), married (324, 91.5%), traders (136, 38.5%), and of the Christian religion (289, 81.8%). Some patients (18, 5.1%) reported a family history of breast cancer. About half of the patients (188, 55.3%) were premenopausal and 144 (42.4%) were postmenopausal. The menopausal status was not known in 8 (2.3%) patients. The mean age at menarche was 14.9 (2.2) years while the mean body mass index was 24.01 (4.0) kg/m2.

**Table 1:** Selected characteristics of breast cancer patients who had mastectomy in Surgical Oncology Division, UCH Ibadan in 1999–2009

**Characteristic**	**Mean (SD)**	**No (n = 354)**	**%**
Age (years)	47.4 (11.3)		
Sex			
Male		14	3.8
Female		340	96.2
Ethnicity			
Yoruba		258	73.0
Ibo		66	18.7
Others		30	8.3
Religion			
Christianity		289	81.8
Islam		62	17.6
Others		3	0.6
Marital status			
Married		324	91.5
Single		22	6.1
Others		8	2.4
Occupation			
Student		15	4.3
Housewife		18	5.2
Trader		136	38.5
Professional		83	22.9
Others		102	29.1
Family history of breast cancer		18	5.1
Age at menarche	14.9 (2.20)		
Parity	3.0 (2.0)		
Menopausal status			
Premenopausal		188	53.1
Postmenopausal		144	42.4
Not known or applicable		8	2.3
Height (cm)	162 (7.1)		
Weight (kg)	63.0 (15.3)		
Body mass index (kg/m2)	24.01 (4.0)		

SD, Standard deviation.

### Clinical and pathology information

Table 2 shows the clinical and pathological characteristics of the patients. The duration of breast lump before presentation at the UCH Ibadan ranged from 1 to 84 months with a mean (SD) of 12.3(14.4) months. Breast cancer occurred in the right breast in 184 (52.2%) patients while left sided and bilateral tumors were found in 164 (46.4%) and 6 (1.4%) patients respectively. Clinically, axillary node involvement was seen in 189 (53.4%) patients, absent in 89 (25.1%) and not stated in 76 (21.5%) patients. The patients were grouped into disease stages as follows: stage I, 89 (25.0%); stage II, 188 (55.4%); stage III, 28 (7.8%); and stage IV, 49 (13.8%).

**Table 2:** Clinico-pathological characteristics of breast cancer patients who had mastectomy in Surgical Oncology Division, UCH Ibadan in 1999–2009

**Characteristic**	**Mean (SD)**	No^*^	**%**
Duration of breast lump (months)	12.3 (14.4)		
Tumour side			
Right		184	52.2
Left		164	46.4
Both		6	1.4
Axillary lymph nodes involvement			
Yes		189	53.4
No		89	25.1
Not stated		76	21.5
Distant metastasis		49	17.1
Site of metastasis (n = 49)			
Lung		25	52.2
Spine		11	21.7
Liver		7	15.2
Other bones		6	10.9
Histology type			
Invasive ductal		249	70.3
Others		105	29.7
Immunochemistry ER (n = 185)			
Positive		110	59.5
Negative		75	40.5
Immunochemistry PR (n = 185)			
Positive		97	52.4
Negative		88	47.6
Immunochemistry Her 2 neu (n = 185)			
Positive		38	20.5
Negative		147	79.5

SD, Standard deviation; * n = 354 unless otherwise stated.

The main sites of metastasis were lung (25, 7.0%), spine (11, 3.1%), liver (7, 2.0%) and other bones (6, 1.7%). Invasive ductal carcinoma was the commonest histological type in 249 (70.3%) patients. Immunohistochemistry results were available for the tumors of 185 (52.3%) patients. Of these, 110 (59.5%) were estrogen receptor (ER) positive, 97 (52.4%) were progesterone receptor (PR) positive and 38 (20.5%) were HER 2 positive.

### Adjuvant treatment

**Figure 2:**
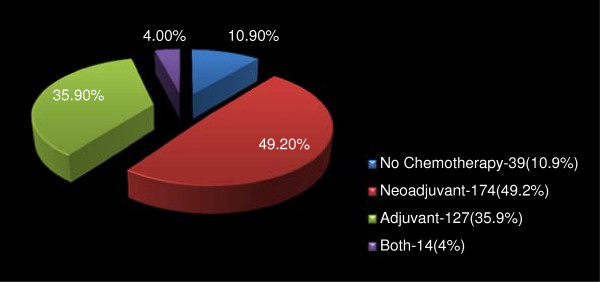
Distribution of administration of chemotherapy in 354 patients who had mastectomy in Surgical Oncology Division, UCH Ibadan in 1999–2009.

**Figure 3:**
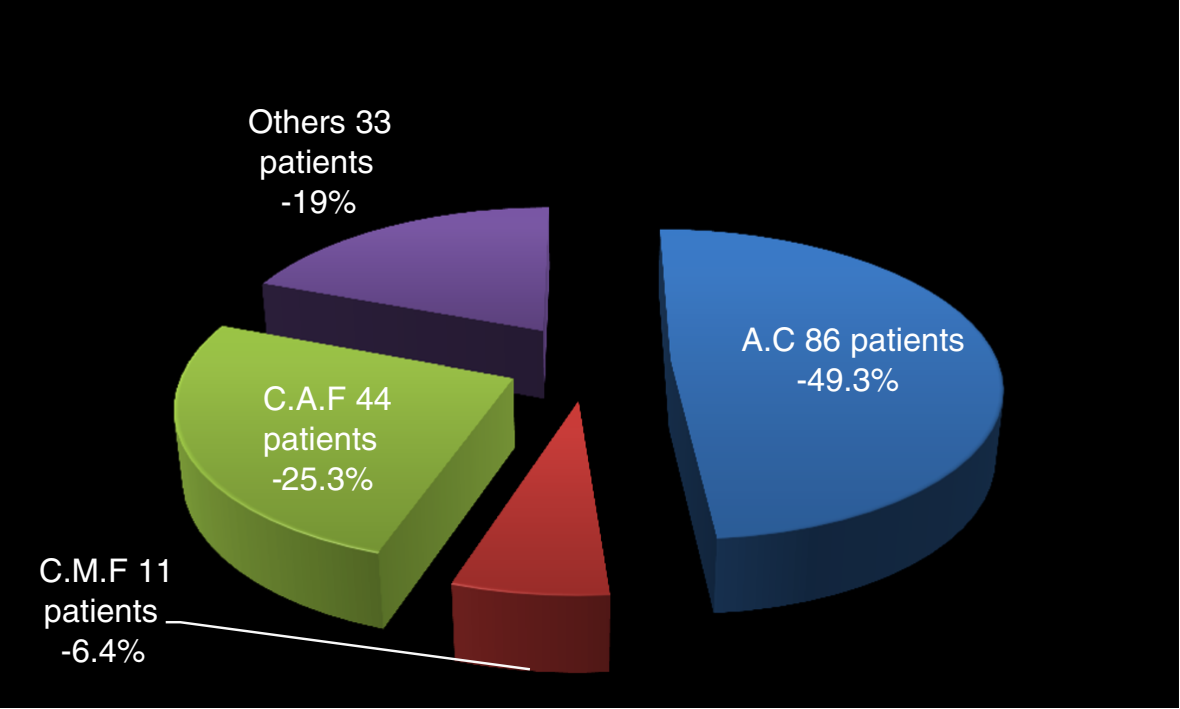
Neo-adjuvant chemotherapy regimens in 174 pre-mastectomy patients in Surgical Oncology Division, UCH Ibadan in 1999–2009.

**Figure 4:**
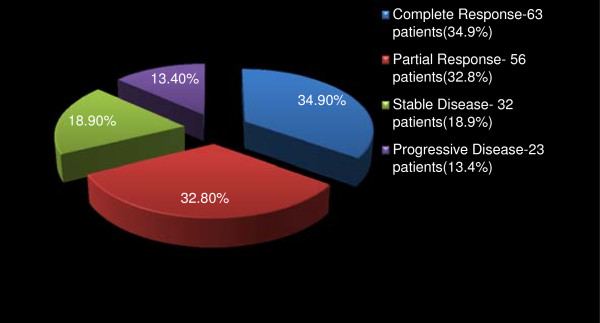
Tumour responses to neo-adjuvant chemotherapy in 174 pre-mastectomy patients in Surgical Oncology Division, UCH Ibadan in 1999–2009.

Altogether, 315 (89.0%) patients received anti-cancer chemotherapy. Of these, 174 (49.1%) patients were treated in the neo-adjuvant setting, 127 (35.9%) post mastectomy and 14 patients (4.0%) both pre and post-mastectomy (Figure 2). Figures 3 and 4 respectively depicts the drug regimens and tumor response in the 174 patients that received neo-adjuvant combination chemotherapy. The anticancer drugs were sourced mainly from the hospital pharmacy and most of the patients completed 4 to 6 courses depending on the regimen used. Payment for the drugs was out-of-pocket and the choice of regimen was dictated mainly by cost and affordability by the patients. Of the 174 patients, 63 and 56 had complete and partial clinical response respectively, amounting to an overall clinical response rate of 67.7%. Tumour dimensions were assessed using caliper and hand measurements, and these were corroborated with the histological reports of mastectomy specimens in some cases. The commonly administered chemotherapy regimens consisted of standard doses of CMF, CAF, and AC and tumor response rate by drugs is as shown in Table 3. Most of the patients received AC regime because of the shift in the unit’s policy sometimes during the period of this review from a combination of CAF to AC. Further to surgery and chemotherapy, 212 (67.0%) and 119 (37.7%) patients received adjuvant radiotherapy and hormonal therapy respectively. Hormonal therapy consisted mainly of daily dose of 20 mg tamoxifen for at least 2 years.

**Table 3:** Responses to neo-adjuvant chemotherapy regimens in 174 pre-mastectomy patients in Surgical Oncology Division, UCH Ibadan in 1999-2009

	**Complete response**	**Partial response**	**Stable disease**	**Disease progression**	**Total (%)**
AC	34 (18.8%)	29 (16.9%)	16 (9.5%)	7 (4.1%)	86 (49.3)
CAF	16 (8.8%)	11 (6.5%)	11 (6.5%)	6 (3.5%)	44 (25.3)
CMF	4 (2.3%)	5 (2.9%)	-	2 (1.2%)	11 (6.4)
Others.	9 (5.0%)	11 (6.5%)	5 (2.9%)	8 (4.6%)	33 (19.0)
Total (%)	63 (34.9%)	56 (32.8%)	32 (18.9%)	23 (13.4%)	174 (100)

A, Adriamycin; C, Cyclophosphamide; F, 5-Fluorouracil; M, Methotrexate.

### Surgical treatment

Modified radical mastectomy was the surgical treatment in 342 (96.7%) patients (Table 4). This consisted of excising the whole breast tissue off the chest wall including the axillary tail and complete axillary clearance of lymph nodes and connective tissue up to level 3 by generous retraction of the pectoralis minor muscle. Where involved, a portion of the underlying muscle was excised in continuity with the breast tissue. A few patients, 7 (2.1%), were offered quadrantectomy and axillary clearance. The type of surgical procedure performed was not stated in 5 (1.2%) patients. The mode of anesthesia was general anesthesia in 293 (82.7%) patients, local infiltration in 11 (3.0%) patients and not stated in 50 (14.3%) patients. Most mastectomies, 239 (67.5%), were done by consultant surgeons while the rest were performed by resident surgeons. About a quarter of the patients received perioperative blood transfusion, 53 (14.9%) during surgery and 35 (10.0%) post-operatively. All the patients transfused were those with either T3 or T4 tumors at presentation and the mean number of blood units transfused was 1.9 units. The mastectomy wounds were drained using close passive drain in 315 (88.7%) patients, close active (suction) drain in 19 (5.4%) patients and unknown in 20 (5.9%) patients.

**Table 4:** Surgical and peri-operative care of mastectomy patients in Surgical Oncology Division, UCH Ibadan in 1999–2009

**Characteristic**	No^*^	**%**
Type of mastectomy		
Modified radical mastectomy	342	96.7
Quadrantectomy	7	2.1
Not stated	5	1.2
Mode of anaesthesia		
General anaesthesia	293	82.7
Local infiltration	11	3.0
Not stated	50	14.1
Cadre of Surgeon		
Consultant	239	67.5
Resident	115	32.5
Blood transfusion (n = 88)		
Intraoperative	53	14.9
Postoperative	35	10.0
Mean number of units	1.9	
Types of drain		
Passive	315	88.7
Suction	19	5.4
Unknown	20	5.9
Postoperative complications (n = 51)		
Seroma	21	6.0
Wound infection	16	4.4
Flap necrosis	6	1.7
Others	8	2.3
Mean hospital stay (days)	12	
Follow up at 18 months post mastectomy		37.9

*n = 354 unless otherwise stated.

### Post-operative care and follow up

**Figure 5:**
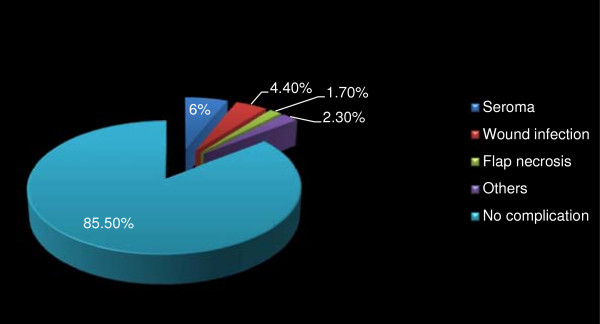
Complications of mastectomy in 51/354 breast cancer patients in Surgical Oncology Division, UCH Ibadan in 1999–2009.

Post-operative complications within 30 days of surgery occurred in 51 (14.5%) patients with seroma accumulation under the skin flap ranking highest in 21 (6.0%) of the patients. Surgical wound infection occurred in 16 (4.4%) and skin flap necrosis in 6 (1.7%) patients. Other less common post-operative events in 8 (2.3%) patients included shoulder joint stiffness and paraesthesia of the upper arm (Figure 5). The surgical drains were removed between the 8th and 14th postoperative day with a mean of 10 days. The wound drains were removed when the output was clear or straw coloured and less than 40mls in the preceding 24 hr period. None of the patients was discharged home with the drain. The least and maximum duration of hospital stay from admission for mastectomy to discharge post-operatively was 9 days and 15 days with a mean (SD) of 12 days. Most patients were seen at the surgical outpatients department for follow up care for less than 2 years. At 18 months, 37.9% of the patients were still attending the outpatients clinics, followed thereafter by a gradual decline with most patents lost to follow up between the 24th and 30th months post mastectomy.

## Discussion

In this review, most patients were young women, premenopausal, had invasive ductal carcinoma and underwent modified radical mastectomy as the definitive surgical treatment. Prior to mastectomy, the tumor was down staged in half of the patients that received neo-adjuvant combination chemotherapy. About two-thirds and a third of all the patients received post-operative radio- and hormonal therapy respectively. The most frequent operative complication was seroma collection in six percent of the patients. The average hospital stay was ten days and most patients were followed up at the surgical outpatients department for about two years post surgery.

Surgery remains an important and essential component of the management of breast cancer, the increasing availability and utilization of adjuvant therapies notwithstanding. In the period under review however, only about one third of the patients with breast cancer received surgical treatment. This breast cancer surgery (BCS) rate was low compared to findings from a similar review in New Zealand where 58.6% of their patients over a 5 year period had BCS [7]. The reasons that possibly contributed to the low mastectomy rate in our patients include late presentation with inoperable local or metastatic tumours necessitating only palliative or terminal care, inability to pay out-of-pocket for treatment, and unwillingness to have mastectomy with consequent loss to follow up [9].

The pattern of clinical presentation and histological types seen in these patients were similar to earlier reports from Nigeria and the West African sub-region [3,10-12]. Breast cancer in many developing countries is characterized by late presentation, younger age at diagnosis, large tumours and multiple nodal involvements. A recent survey in Lagos, a nearby city showed that more than four-fifths of the patients delayed for 3 months before the initial medical consultation [13]. The pattern of presentation differs from that in the western world where most patients are post-menopausal and present with small sized early tumors and less aggressive disease. This is more a reflection of the differences in the demographic pattern of these societies than of assumed differences in the intrinsic biology of the disease in the two populations [10,14,15]. The predominance of invasive ductal carcinoma is also akin to what is seen in other parts of the world.

Although immunohistochemistry was obtained in only half of these patients, its pattern nonetheless, tallies with what has been previously reported from our group in the same cohort of patients [16]. About two-thirds of the tumors had either estrogen or progesterone receptor positivity and one-fifth of them were Her2 neu positive. Previous immunohistochemistry reports had suggested that breast cancer tumors in Africans were estrogen or progesterone receptor poor [17,18]. These findings were unreflective of the true picture given that those studies were done on old archival tissues in which antigen degradation had occurred [19,20]. Our findings are similar to values from other populations, including from within Africa, and further support our earlier conclusion that the pattern of hormone receptors in breast cancer patients of African origin does not differ from others [16,21-23].

More than half of our patients with locally advanced cancer had positive tumour response and down staging by the use of adjuvant chemotherapy. This supports the role of neo adjuvant chemotherapy in our patients in whom one in every two patients with advanced disease can be down staged thus enhancing the prospects of loco-regional surgical tumor extirpation. A previous report from Eastern Nigeria had documented a response rate of 81% in 32 women with locally advanced breast cancer who received neo-adjuvant combination CAF chemotherapy [24]. Since its inception in the early 1970s, systemic chemotherapy as an adjunct to surgery has emerged as the foremost conceptual change in the treatment of breast cancer [25]. It also improves overall survival in both younger and older women with node positive breast cancer [26]. Anthracycline based combination chemotherapy was the most commonly used regimen in our patients, and this had been shown to reduce primary tumour burden by at least 50% as far back as the pre-taxanes era [25]. Moreover, our patients received adjuvant radiation and hormonal therapies as indicated as part of standard anti-cancer treatment. Radiation therapy consisted mainly of post mastectomy chest wall irradiation and irradiation of localized secondary deposits in the spine, in long bones and the brain when indicated. Documentation problems in patients’ case files most probably account for the apparently low rate of hormonal therapy in the patients.

The surgical option in almost all patients was modified radical mastectomy (MRM). This still remains the standard surgical operation for operable breast cancer in most parts of the world. The indications for MRM in our patients were early breast cancer (stages I and II), locally advanced disease pre- or post-adjuvant treatment and in select patients with metastatic disease who required local control post systemic therapy. Only a negligible number of our patients had breast conserving surgery (BCS). Early presentation and the availability and extensive use of adjuvant therapies have made BCS to gain widespread usage in some parts of the world [27,28]. This is especially so in developed countries where evidence now abounds that it is as effective as MRM in early breast cancer [2,29]. Though a feasible option in some patients, BCS is likely to remain an infrequent surgical treatment for breast cancer in many low income settings for a long time to come. This is because most patients present late for treatment and more often with poor grade tumours. Moreover, there are limitations that are imposed by logistics such as paucity of radiotherapy services and inefficient follow-up programmes. The inability of patients to follow through post-surgery treatment schedules due to low literacy levels and poverty contribute to these challenges. Although sentinel lymph node (SLN) biopsy has become the preferable standard to axillary dissection in breast cancer surgery, this was not done in any of our patients. Going forward from this review, and with the requisite expertise acquired, new patients that present with no palpable lymph nodes and those with good response to neo-adjuvant chemotherapy will be considered for this procedure. This would hopefully reduce needless axillary clearance and its attendant risks and complications.

Almost a quarter of the patients received blood transfusion either during surgery or in the post-operative period and transfusion occurred only in those with locally advanced tumours. Perioperative blood transfusion in breast cancer patients had been a subject of study in the past decade with some suggesting that blood transfusion had harmful effects on outcome [30-32] and others failing to support such notion [33,34]. Perioperative blood usage was high in our patients. This contrasts with a 2 year British hospital audit spanning 2004/5 in which the transfusion rate was 3.8% for mastectomy and 20.0% for mastectomy with immediate breast reconstruction [35]. Though not stated in that report, the clinico-pathological pattern and prognostic indicators in their patients were likely to be more favourable given that most of them were likely to present early for treatment. The use of blood transfusion in the first and second halves of our study period did not show a remarkable difference. However, in our current experiences perioperative blood usage is significantly minimal. This may partly be due to our current standard use of neo-adjuvant treatment to downstage the tumor in locally advanced breast cancer.

Ezeome and Adebamowo had earlier reported that closed simple drains were cheap, simple to manage and effectively comparable to closed suction drains for mastectomy wounds [36]. These characteristics make closed passive drain the more attractive option for breast cancer surgery in low income countries and explain why this was predominantly used in our patients. In the study by Ezeome and Adebamowo, a higher wound infection rate was associated with passive tube drains compared to suction drains. In our current review, however, the infection rate was similar to what had been reported from other studies where closed suction drains were predominantly used [32,37]. Akin to the discussion on types of drain is consideration of the length of drainage and the duration of in-patient care after mastectomy. The length of drainage seems to be directly proportional to the duration of postoperative in-hospital care. The standard management of mastectomy drain was to remove the drain when drainage was minimal, usually less than 40 ml over a 24-hour period or after 12–14 days whichever came first [37]. This protocol kept the patient in hospital for an average of 8 to 10 days and increased the hospital bill significantly. Some studies compared this protocol with a policy of either no drain at all or early drain removal and early postoperative discharge from the hospital. The results recorded were shorter hospital stay, no difference in surgical or psychological morbidity and significant cost savings [37-39]. Our drain policy is still evolving but the present practice is to admit patients on the day before surgery, review their wound on the 5th postoperative day and discharge them as soon as the drain can be removed thereafter.

The overall morbidity and the rates of postoperative events in our cohort compare favorably with what have been variously reported in literature. Seroma accumulation under the skin flap was the commonest complication as has been documented in many series [8,40-42]. Factors that have been associated with seroma formation include large dead space, irregularity of chest wall, low concentration of endostatin (an anti-angiogenic factor), use of electrocautery for flap dissection compared to scalpel and modified radical mastectomy compared to breast conserving mastectomy [8,42]. In a pooled analysis of prospectively enrolled 3107 women who had mastectomy in 18 hospitals over a 3 year period, Et-Tamer et al. reported an overall wound infection rate of 4.3%.^8^ The rate of infection in our series tallies closely with theirs. Moreover, mastectomy was associated with more wound issues than conservative surgery in their series. A little above a third of the patients were attending the surgical outpatients department 24 months after mastectomy. The reasons for loss of many to long time follow up are not known, thus the survival pattern in this cohort might never be determined. However, a previous Nigerian study reported a median survival of 31 months with survival advantage for post-menopausal women and in those with early stage disease [43].

## Conclusions

Modified radical mastectomy remains the standard therapeutic surgical operation for breast cancer in Nigeria. Though many patients present late for treatment, tumor down-staging with neo-adjuvant chemotherapy enhances surgical loco-regional tumor control in some of them. The overall morbidity and the rates of postoperative events are minimal and comparable to what obtain in other parts of the world. However, long-term follow-up is still a challenge as many patients are lost to follow up after two years of mastectomy. This review has limitations due to its retrospective nature. The case notes of some of the patients that had mastectomy were not available for review as at the time of the study. Moreover some data were missing from some case notes such that the denominator was not uniform for all the variables that were analyzed. Furthermore, the pattern of survival of the patients post-mastectomy cannot be determined because of inadequate follow up records and information.

## Competing interests

The authors declare that they have no competing interests.

## Authors’ contributions

TO participated in patients’ management, conceived the study, coordinated and co-wrote the manuscript. OA participated in patients’ management, study design and co-wrote the manuscript. AA participated in patients’ management and study design. CA participated in patients’ management, study design and co-wrote the manuscript. All authors read and approved the final manuscript.

## Pre-publication history

The pre-publication history for this paper can be accessed here:

http://www.biomedcentral.com/1471-2482/13/59/prepub
